# Health care utilization among Medicare-Medicaid dual eligibles: a count data analysis

**DOI:** 10.1186/1471-2458-6-88

**Published:** 2006-04-05

**Authors:** Sangho Moon, Jaeun Shin

**Affiliations:** 1 Department of Public Administration, Sungkyunkwan University, Seoul, Korea; 2 KDI School of Public Policy and Management, Seoul, Korea

## Abstract

**Background:**

Medicare-Medicaid dual eligibles are the beneficiaries of both Medicare and Medicaid. Dual eligibles satisfy the eligibility conditions for Medicare benefit. Dual eligibles also qualify for Medicaid because they are aged, blind, or disabled and meet the income and asset requirements for receiving Supplement Security Income (SSI) assistance. The objective of this study is to explore the relationship between dual eligibility and health care utilization among Medicare beneficiaries.

**Methods:**

The household component of the nationally representative Medical Expenditure Panel Survey (MEPS) 1996–2000 is used for the analysis. Total 8,262 Medicare beneficiaries are selected from the MEPS data. The Medicare beneficiary sample includes individuals who are covered by Medicare and do not have private health insurance during a given year. Zero-inflated negative binomial (ZINB) regression model is used to analyse the count data regarding health care utilization: office-based physician visits, hospital inpatient nights, agency-sponsored home health provider days, and total dental visits.

**Results:**

Dual eligibility is positively correlated with the likelihood of using hospital inpatient care and agency-sponsored home health services and the frequency of agency-sponsored home health days. Frequency of dental visits is inversely associated with dual eligibility. With respect to racial differences, dually eligible Afro-Americans use more office-based physician and dental services than white duals. Asian duals use more home health services than white duals at the 5% statistical significance level. The dual eligibility programs seem particularly beneficial to Afro-American duals.

**Conclusion:**

Dual eligibility has varied impact on health care utilization across service types. More utilization of home healthcare among dual eligibles appears to be the result of delayed realization of their unmet healthcare needs under the traditional Medicare-only program rather than the result of *over*utilization in response to the expanded benefits of the dual eligibility program. The dual eligibility program is particularly beneficial to Asian and Afro-American duals in association with the provision of home healthcare and dental benefits.

## Background

It has been an important financial issue in the Medicare system that health care expenses of dually eligible beneficiaries (DEB) are much higher than those of Medicare-only beneficiaries (MOB). Total health expenditures for the dually eligible beneficiaries are more than double those of the Medicare-only beneficiaries. In 1999, total annual health expenditures (including Medicare, Medicaid, private, and out-of-pocket spending) averaged $16,278 for each dually eligible beneficiary, compared with $7,396 on average for those who are not dually eligible [1].

Dually eligible beneficiaries, who are covered by both Medicare and Medicaid, represent only one-fifth of each program's enrollment, about 7 million in 1997, but account for a much larger share of each program's spending (Komisar et al., 2000) [2]. In 1999, these dually eligible beneficiaries accounted for about $50 billion in Medicare expenditures (24 percent of total Medicare spending) and $63 billion in Medicaid expenditures (35 percent of total Medicaid spending) nationwide, reflecting their relatively greater medical and long-term care demands [1]. The dually eligible beneficiaries are the most costly population being served by publicly funded health care programs [3].

The dually eligible population are more likely to be disabled and either younger (under age 65) or older (over age 85) than the majority of Medicare beneficiaries [3]. Over half of the dually eligible beneficiaries are in fair or poor health, whereas only one quarter of the entire Medicare beneficiaries are reported to be in fair or poor health. In particular, the dually eligible beneficiaries are more likely to suffer from chronic and serious health conditions such as diabetes, pulmonary disease, and stroke. More than 40 percent of dually eligible beneficiaries have a cognitive or mental impairment, while only 9 percent of the entire Medicare population have similar mental problems [1,4]. Dually eligible beneficiaries are culturally diverse. Over 42 percent of the dual eligibles represent racial minority population, whereas 16 percent of the entire Medicare beneficiaries belong to the racial minority groups [3].

Known for the high costs and complex healthcare needs, dually eligible beneficiaries have been the center of debate in both programs as neither Medicare nor Medicaid take full responsibility to face the medical needs of the dual eligibles. Dually eligible beneficiaries are still viewed as a heavy liability to public and private insurers in the United States [3]. In spite of its importance in establishing an efficient and reliable public healthcare system, studies on the dual eligibility program are limited.

The purpose of this study is to examine the health care utilization by dually eligible beneficiaries compared with Medicare-only beneficiaries. Medicare-Medicaid dual eligibles have more benefits by virtue of also being enrolled in both programs. We will explore the effect of the additional Medicaid coverage on their healthcare utilization with special emphasis on racial differences. We categorized the sample population into four racial groups: Whites, Asians, Hispanics, and Afro-Americans. Hispanics indicate those who self-identified themselves from Hispanic origin including both whites and non-whites.

We limit our sample to individuals who have Medicare coverage continuously throughout a given year but have never any private health insurance. By controlling for demographic, socioeconomic, and health-related potential confounding factors, we estimate the magnitude of the statistical association between dual eligibility and the frequency/likelihood of using healthcare services. We employ a zero-inflated negative binomial (ZINB) regression model to incorporate statistical features of the count data as demonstrated in the Methods section.

## Methods

### Data

We used data from the 1996–2000 waves of the Medical Expenditure Panel Survey (MEPS). The MEPS is a nationally representative survey conducted by the U.S. Agency for Healthcare Research and Quality (AHRQ) and the National Center for Health Statistics (NCHS). This ongoing survey collects detailed information on health status, healthcare use, medical expenditures, and insurance coverage as well as various socioeconomic and demographic characteristics for the U.S. civilian and non-institutionalized population.

Medicare beneficiaries are the population under study. During 1996–2000, the MEPS interviewed 130,938 individuals; among them, 8,262 individuals are selected on the basis of self-reporting that they have continuous Medicare coverage for the entire calendar year. Individuals with private health insurance at any time as supplemental to Medicare were excluded to avoid contamination from the effect of Medicare-private dual eligibility. Approximately, 22.8% of the sample population, i.e., 1,181 individuals out of the 8,262 Medicare sample beneficiaries, were identified as Medicare-Medicaid dual eligibles.

### Dependent variables

Utilization variables are measured as the total number of office-based physician visits, hospital inpatient nights, agency-sponsored home health days, and dental visits per year.

Office-based physician visits consist of encounters with physician services that took place primarily in office-based and clinic settings. Health services provided in other settings such as a hospital, nursing home, or a person's home do not belong to this category. Also, non-physician visits to chiropractors, midwives, nurses and nurse practitioners, optometrists, podiatrists, physician's assistants, physical therapists, occupational therapists, psychologists, social workers, technicians, receptionists/clerks/secretaries, or other medical providers are not included in this category. The analysis focuses on the number of visits to physicians because the majority of office-based visits are concentrated on physician providers.

Hospital inpatient nights are measured by the number of nights spent in hospitals for receiving inpatient care. This variable is chosen for examining the utilization of hospital inpatient services by patients.

Agency-sponsored home health provider days indicate the number of days in which home healthcare services are provided by paid caregivers such as hospitals or nursing homes. Information on the utilization of home healthcare is collected by the MEPS on a monthly basis. By adding up the number of provider days per month for all home health providers seen, the number of home health provider days per year is obtained. For example, if a person received care in one month from one provider on two different days, the number of provider days would equal two. If a person received care from two different providers on the same day, the number of provider days would also equal two. However, if a person received care from one provider two times in the same day, then the provider days would equal one.

Total dental visits represent the total number of dental visits per year to any dental care provider(s) including general dentists, dental hygienists, dental technicians, dental surgeons, orthodontists, endodontists, and periodontists.

### Explanatory variables

Table 1 documents the descriptive statistics of the sample for the analysis. The average age of the sample is 69.3. Among the sample members, 41% are male; 41% are married; 27% live in rural areas; 48% have education of less than high school graduation.

Hispanics and Afro-Americans represent 17% and 18% of the sample, respectively. Approximately 22.8% of the sample members are Medicare-Medicaid dually eligible beneficiaries. One out of four individuals is in poverty and almost one out of ten members works as a part-time/temporary worker.

According to the self-reported health indicator, 36% of individuals report that they are in fair or poor health out of five-scale measurement. Approximately, 48% of the respondents have chronic medical conditions such as diabetes, cancer, hypertension, heart failure, arthritis, depression, and other mental disorders. Respondents on average have 5.67 co-morbid symptoms.

### Statistical analysis

Measures for healthcare use (denoted by *y*) have three fundamental statistical properties: (1) to be non-negative (*y *≥ *0*); (2) to have a non-trivial fraction of zero outcomes; and (3) to follow a positively skewed distribution of the non-zero realizations. These unique count data structures enforce our empirical investigation to rely on the count model as illustrated below:

#### Model 1

Generalized linear model (GLM) relates an outcome (*y*) to exogenous covariates *x *as follows:

*g*(*E*(*y*)) = *xβ*, *y *~ *F *    (1)

where *g(.) *is called the link function and *F *refers to the distributional families. Substituting various specifications for *g(.) *and *F *result in an array of the models. For instance, if we assume *F *to be a Poisson or negative binomial (NB) distribution, it gives us a log-linear model of the count data:

log(*E*(*y*)) = *xβ*, *y*~*Poisson/Negative Binomial (NB) *    (2)

Poisson regression assumes that the dependent variable *y*, the number of occurrence of an event, has a Poisson distribution given an independent variable vector *x*,

P(*y *= *k*| *x*) = e^-*μ *^*μ*^k ^/ k!,     k = 0, 1, 2, ......,     (3)

It is easily shown that the mean and variance of this distribution equal to *μ*, or *E*(*y*) = var(*y*) = *μ*. Then, equation (2) can be written as a log-linear function of the independent variables *x *given as

log(*μ*) = *xβ *or equivalently, *μ *= exp(*xβ*).     (4)

The maximum likelihood method is used to estimate the parameters of a Poisson regression model. From equation (4), the log-likelihood function is given as

log⁡L(β)=∑i{yilog⁡(μi)−μi},     (5)
 MathType@MTEF@5@5@+=feaafiart1ev1aaatCvAUfKttLearuWrP9MDH5MBPbIqV92AaeXatLxBI9gBaebbnrfifHhDYfgasaacH8akY=wiFfYdH8Gipec8Eeeu0xXdbba9frFj0=OqFfea0dXdd9vqai=hGuQ8kuc9pgc9s8qqaq=dirpe0xb9q8qiLsFr0=vr0=vr0dc8meaabaqaciaacaGaaeqabaqabeGadaaakeaacyGGSbaBcqGGVbWBcqGGNbWzcqWGmbatcqGGOaakiiGacqWFYoGycqGGPaqkcqGH9aqpdaaeqbqaaiabcUha7jabdMha5naaBaaaleaacqWGPbqAaeqaaOGagiiBaWMaei4Ba8Maei4zaCMaeiikaGIae8hVd02aaSbaaSqaaiabdMgaPbqabaGccqGGPaqkcqGHsislcqWF8oqBdaWgaaWcbaGaemyAaKgabeaakiabc2ha9jabcYcaSaWcbaGaemyAaKgabeqdcqGHris5aOGaaCzcaiaaxMaadaqadaqaaiabiwda1aGaayjkaiaawMcaaaaa@51C5@

where *μ *follows equation (4) for all *i*.

Both Poisson and negative binomial regressions are standard count models used to deal with the number of occurrence of an event. The restrictive condition of the equality of mean and variance in the Poisson distribution (*equidispersion*) is the reason that the negative binomial model is often preferred. The negative binomial regression assumes that the dependent count variable *y *follows a negative binomial distribution instead of a Poisson given an independent variable vector *x*,

P(y=k|x)=Γ(k+1/α)Γ(k+1)Γ(1/α)(αμ)k(1+αμ)k+1/α,k=0,1,2,......,     (6)
 MathType@MTEF@5@5@+=feaafiart1ev1aaatCvAUfKttLearuWrP9MDH5MBPbIqV92AaeXatLxBI9gBaebbnrfifHhDYfgasaacH8akY=wiFfYdH8Gipec8Eeeu0xXdbba9frFj0=OqFfea0dXdd9vqai=hGuQ8kuc9pgc9s8qqaq=dirpe0xb9q8qiLsFr0=vr0=vr0dc8meaabaqaciaacaGaaeqabaqabeGadaaakeaafaqabeqacaaabaGaeeiuaaLaeiikaGIaemyEaKNaeyypa0Jaem4AaSMaeiiFaWNaemiEaGNaeiykaKIaeyypa0ZaaSaaaeaacqqHtoWrcqGGOaakcqWGRbWAcqGHRaWkcqaIXaqmcqGGVaWliiGacqWFXoqycqGGPaqkaeaacqqHtoWrcqGGOaakcqWGRbWAcqGHRaWkcqaIXaqmcqGGPaqkcqqHtoWrcqGGOaakcqaIXaqmcqGGVaWlcqWFXoqycqGGPaqkaaWaaSaaaeaacqGGOaakcqWFXoqycqWF8oqBcqGGPaqkdaahaaWcbeqaaiabdUgaRbaaaOqaaiabcIcaOiabigdaXiabgUcaRiab=f7aHjab=X7aTjabcMcaPmaaCaaaleqabaGaem4AaSMaey4kaSIaeGymaeJaei4la8Iae8xSdegaaaaakiabcYcaSaqaaiabdUgaRjabg2da9iabicdaWiabcYcaSiabigdaXiabcYcaSiabikdaYiabcYcaSiabc6caUiabc6caUiabc6caUiabc6caUiabc6caUiabc6caUiabcYcaSiaaxMaacaWLjaWaaeWaaeaacqaI2aGnaiaawIcacaGLPaaaaaaaaa@733B@

where *α *is a dispersion parameter, measuring the extent of *over*dispersion. From equation (6), the log-likelihood function for the negative binomial maximum likelihood estimation is obtained as

log⁡L(μ)=∑iliwhereli=yiln⁡(αμi)−(yi+1/α)ln⁡(1+αμi)+log⁡(Γ(yi+1/α)Γ(yi+1)Γ(1/α))     (7)
 MathType@MTEF@5@5@+=feaafiart1ev1aaatCvAUfKttLearuWrP9MDH5MBPbIqV92AaeXatLxBI9gBaebbnrfifHhDYfgasaacH8akY=wiFfYdH8Gipec8Eeeu0xXdbba9frFj0=OqFfea0dXdd9vqai=hGuQ8kuc9pgc9s8qqaq=dirpe0xb9q8qiLsFr0=vr0=vr0dc8meaabaqaciaacaGaaeqabaqabeGadaaakeaafaqabeqadaaabaGagiiBaWMaei4Ba8Maei4zaCMaemitaWKaeiikaGccciGae8hVd0MaeiykaKIaeyypa0ZaaabuaeaacqWGSbaBdaWgaaWcbaGaemyAaKgabeaaaeaacqWGPbqAaeqaniabggHiLdaakeaacqqG3bWDcqqGObaAcqqGLbqzcqqGYbGCcqqGLbqzaeaacqWGSbaBdaWgaaWcbaGaemyAaKgabeaakiabg2da9iabdMha5naaBaaaleaacqWGPbqAaeqaaOGagiiBaWMaeiOBa4MaeiikaGIae8xSdeMae8hVd02aaSbaaSqaaiabdMgaPbqabaGccqGGPaqkcqGHsislcqGGOaakcqWG5bqEdaWgaaWcbaGaemyAaKgabeaakiabgUcaRiabigdaXiabc+caViab=f7aHjabcMcaPiGbcYgaSjabc6gaUjabcIcaOiabigdaXiabgUcaRiab=f7aHjab=X7aTnaaBaaaleaacqWGPbqAaeqaaOGaeiykaKIaey4kaSIagiiBaWMaei4Ba8Maei4zaC2aaeWaaeaadaWcaaqaaiabfo5ahjabcIcaOiabdMha5naaBaaaleaacqWGPbqAaeqaaOGaey4kaSIaeGymaeJaei4la8Iae8xSdeMaeiykaKcabaGaeu4KdCKaeiikaGIaemyEaK3aaSbaaSqaaiabdMgaPbqabaGccqGHRaWkcqaIXaqmcqGGPaqkcqqHtoWrcqGGOaakcqaIXaqmcqGGVaWlcqWFXoqycqGGPaqkaaaacaGLOaGaayzkaaaaaiaaxMaacaWLjaWaaeWaaeaacqaI3aWnaiaawIcacaGLPaaaaaa@8C84@

Since the standard Poisson model does not allow the presence of *over*dispersion, we employ likelihood ratio (LR) test of the *over*dispersion parameter *α *to examine the validity of the Poisson specification against the negative binomial model.

#### Model 2

The zero-inflated (ZI) count model is a mixing specification that adds extra weight to the probability of observing a zero [5,6]. This can be interpreted as a splitting mechanism that divides individuals into non-users, with probability *ω*, and potential users, with probability 1 - *ω*. We assume that there is a parent distribution function, denoted by *φ*, for outcome realizations. In one regime [Part 1], outcomes are always zero. In the second regime [Part 2], outcomes are non-zeros. For each regime, the probability density function is a weighted average of the parent density given as

*φ*_1 _= *ω *+ (1 - *ω*)*φ*, [Part 1]     (8)

and

*φ*_2 _= (1 - *ω*)*φ*, [Part 2]     (9)

where *ω *is a weight parameter to let the integral of equations (8) and (9) be equal to one. The log-likelihood function Λ is specified from *φ*_1 _and *φ*_2 _as follows:

Λ=∑zeroslog⁡{ω+(1−ω)φ}+∑nonzeroslog⁡{(1−ω)φ}.     (10)
 MathType@MTEF@5@5@+=feaafiart1ev1aaatCvAUfKttLearuWrP9MDH5MBPbIqV92AaeXatLxBI9gBaebbnrfifHhDYfgasaacH8akY=wiFfYdH8Gipec8Eeeu0xXdbba9frFj0=OqFfea0dXdd9vqai=hGuQ8kuc9pgc9s8qqaq=dirpe0xb9q8qiLsFr0=vr0=vr0dc8meaabaqaciaacaGaaeqabaqabeGadaaakeaacqqHBoatcqGH9aqpdaaeqbqaaiGbcYgaSjabc+gaVjabcEgaNbWcbaGaemOEaONaemyzauMaemOCaiNaem4Ba8Maem4CamhabeqdcqGHris5aOGaei4EaShcciGae8xYdCNaey4kaSIaeiikaGIaeGymaeJaeyOeI0Iae8xYdCNaeiykaKIae8NXdyMaeiyFa0Naey4kaSYaaabuaeaacyGGSbaBcqGGVbWBcqGGNbWzcqGG7bWEcqGGOaakcqaIXaqmcqGHsislcqWFjpWDcqGGPaqkcqWFgpGzcqGG9bqFcqGGUaGlaSqaaiabd6gaUjabd+gaVjabd6gaUjabdQha6jabdwgaLjabdkhaYjabd+gaVjabdohaZbqab0GaeyyeIuoakiaaxMaacaWLjaWaaeWaaeaacqaIXaqmcqaIWaamaiaawIcacaGLPaaaaaa@6B51@

The principal motivation for the zero-inflated count model is that observed data frequently display a higher relative frequency of zeros (*excess zeros*) than standard count models, which is the feature of zero-inflated count model [5,6]. This refers to observing more zeros than is consistent with the standard Poisson or another baseline count model specifications^1^[7]. Vuong test statistics are needed to provide the appropriateness of ZI models against the standard count models^2 ^[8].

#### Specification tests

In our study, the utilization of healthcare is measured as the number of physician visits, hospital inpatient nights, home health provider days, and total dental visits. More than 70% of Medicare beneficiaries reported zero utilization for all types of healthcare services except for physician visits (Table 2). For verifying the hypothesis that a modified count model with *over*dispersion is the adequate specification for the analysis, we implement three specification tests to alternative models; (a) a normality test against *Model 1*, (b) a likelihood-ratio (LR) test for the *over*dispersion parameter *α *in the negative binomial (NB) specification against the Poisson model specification^3 ^[7,9,10], and (c) Vuong test of the standard count model against the zero-inflated count model. The test results reject the assumption of the normal distribution; reject the Poisson specification; and reject the standard count model. We conclude that the zero-inflated negative binomial (ZINB) model, in which the parent distribution is specified to follow a negative binomial distribution as in equation (6), is the best fit for our data. The test statistics are available upon request to the authors.

The ZINB regression expresses the count outcome *y *as a function of various explanatory variables x, including demographic and socioeconomic characteristics, health conditions and insurance coverage status as the following:

*φ*(*y*_*i*_,*θ*| *X*) = *φ*(*y*_*i*_, *B*, *γ*|*Z*_*i*_, *Dual*_*Eligible*_*i*_)     (11)

where *φ *is a negative binomial probability density function. *i *denotes the index for an individual. *y*_*i *_is total number of healthcare use (that is, office-based physician visits, hospital inpatient nights, home health provider days, and dental visits) in a given year for an individual *i*. The dependent count variable *y*_*i *_follows a negative binomial distribution given an independent variable vector *X*_*i*_, where *θ *represents a coefficient vector incorporated with the negative binomial probability density function. *Z*_*i *_represents a set of explanatory variables such as age, gender, marital status, education, race/ethnicity, employment status, family income, region of residence, self-reported health status, number of co-morbidities, limitations to activities, diagnosed chronic diseases and dummies for a survey year. Our major control variable is a binary indicator of *Dual*_*Eligible*_*i*_, which is equal to 1 if an individual *i *is dually eligible and 0 otherwise. *B *and *γ *indicate the corresponding coefficients for *Z*_*i *_and *Dual_Eligible*_*i*_, respectively.

## Results

### Who are dually eligible population?

Table 1 shows differences in demographic and socioeconomic characteristics between Medicare-only beneficiaries (MOB) and dually eligible beneficiaries (DEB). Mean age is 70.9 for Medicare-only beneficiaries while 64.1 for dually eligible beneficiaries. Compared with Medicare-only beneficiaries, the dually eligible beneficiaries are more likely to be female (68% versus 57%), unmarried (77% versus 53%), and less educated (4% versus 13% with college education and more). As to socioeconomic status, probability of being in poverty is much higher among dually eligible beneficiaries (45%) than among Medicare-only beneficiaries (19%).

Regarding health status, the dually eligible beneficiaries are more likely to have adverse medical conditions listed as predetermined priority conditions (priority list) such as hypertension, diabetes mellitus, cardiac problems, and arthritis. On average, 51% of the dually eligible beneficiaries suffer from any priority conditions compared with 48% of Medicare-only beneficiaries to have chronic disease(s). Mean number of co-morbidities among dually eligible beneficiaries is slightly larger (6.47) than that of Medicare-only beneficiaries (5.44). Whereas about 20% of dual eligibles have limitation(s) to activities of daily living (ADL), only about 9% of Medicare-only beneficiaries have difficulties in ADL. Approximately 33% of dually eligible beneficiaries have at least one limitation to instrumental activities of daily living (IADL), compared with 15% of Medicare-only beneficiaries. These findings indicate that dually eligible beneficiaries are more vulnerable to chronic health conditions and frailty. Consequently they have more health care needs than Medicare-only beneficiaries.

Table 3 reports the estimation results for the relationship between dual eligibility and healthcare utilization: office-based physician visits, hospital inpatient nights, agency-sponsored home health provider days, and total dental visits. The likelihood ratio (LR) test statistics of the *over*dispersion parameter *α *indicate that the Poisson model is rejected (and the negative binomial (NB) model is employed) at the 5% level of statistical significance for all outcome variables. Vuong (1989) test results reveal that for all outcome measures the ZINB model is preferred against the standard NB model. As the result of the series of specification tests, the ZINB model is chosen as the best fit to the count data in use.

The covariate effect of the extra-zero component in the model is estimated by a logit regression. The probability of being a non-user of healthcare and estimated coefficients as a logit function of observed covariates are reported in Table 3. In the logit inflation model, the dependent variable is an indicator 1(*y*_*i *_= 0), which takes 1 if *y*_*i *_= 0, and zero otherwise.

Based on the logit results, the dually eligible beneficiaries (DEBs) are more likely to use hospital inpatient care (odd ratio = 1.33 (= exp(0.285)) and home health care (odd ratio = 2.99 (= exp(1.066)) at the 5% level of significance. However, dually eligible beneficiaries (DEBs) are not statistically different from Medicare-only beneficiaries (MOBs) in the likelihood of using office-based physician and dental services at the 5% level of significance.

Columns (1) and (2) of Table 3 indicate that, among the potential users of Medicare beneficiaries including whites, dual eligibility does not have any statistically significant correlation with either office-based physician visits or hospital inpatient nights at the 5% statistical significance level. However, physician visits by Afro-Americans increased owing to dual eligibility in office-based physician services at the 5% level.

Dual eligibility is statistically significant to increase the likelihood of using hospital inpatient care at the 5% level (odd ratio = 1.33 (= exp(0.285)). But it is not statistically associated with the number of hospital inpatient nights among hospital care users at the 5% level. Since the logit result is derived from the entire sample of both non-users and users, the overall relationship between dual eligibility and hospital care utilization may be regarded as positive.

Column (3) shows that dual eligibility is positively correlated with the likelihood of using agency-sponsored home health days (odd ratio = 2.99 (= exp(1.066)) as well as the frequency of service receipt days among the users (IRR = 1.958) at the 5% level of statistical significance.

With respect to racial differences in the utilization of home healthcare, the magnitude of the correlation between the frequency of home healthcare use by Asians and dual eligibility is particularly impressive. In general, Asians utilize home health services less often than whites (IRR = 0.044) whereas Hispanics and Afro-Americans make more frequent visits to home healthcare providers (IRR = 1.790 for Hispanics and IRR = 2.216 for Afro-Americans) than whites. Among Asian duals, however, the level of home healthcare use substantially rises compared to other races with dual eligibility at the 5% level of statistical significance (IRR = 33.05(= 1.958*16.879)).

The statistically significant, large effect of dual eligibility on the frequency of home healthcare receipt days is related to the level and scope differences in home health benefits between Medicare-only and Medicare-Medicaid dual eligibility programs. The home healthcare benefits covered by Medicaid is much more comprehensive than that of Medicare by including many other benefits that might be collectively called long-term care. These benefits include chore aides in case-specific situations and personal care services/attendants, which many states choose to include in their Medicaid programs. All such services except for home care are optional in Medicare, whose coverage and payments for home health services vary from state to state.

As to the scope of home healthcare services provided, Medicaid payments are not restricted to skilled services as are Medicare payments. Also, unlike Medicare, Medicaid home health does not require that an individual be home bound. Medicaid pays for almost all sorts of home healthcare, reducing individual financial burdens effectively. Medicare, however, with its limited payment, often fails to meet its beneficiaries' home healthcare needs. As a result, Medicare-only beneficiaries are forced to pay for needed services out-of-pocket resources or to skip the receipt of services. Our empirical results indicate that expanded home health benefits from Medicaid help Asian duals receive home healthcare, which sometimes are not properly provided by the Medicare-only program.

Column (4) in Table 3 indicates that low education is inversely correlated with both the likelihood of using dental services and the frequency of dental visits at the 5% level. The incidence rate ratio (IRR) of dental visits by dual eligibles indicates that dual eligibles, whose average level of education is lower than that of Medicare-only beneficiaries (as shown in Table 1), are less likely to utilize dental services compared to their non-dual counterparts.

With respect to racial differences, Afro-American dual eligibles are positively associated with the frequency of dental visits at the 5% level (IRR = 1.36 (i.e., 0.708*1.926)). Afro-Americans are correlated with lower frequency of dental visits than whites (IRR = 0.622) significantly at the 5% level, which is quite contrast to the increased frequency of dental visits by Afro-American dual eligibles (IRR = 1.36 (i.e., 0.708*1.926)).

Without dental benefits under the Medicare-only program, Medicare-only beneficiaries are forced to pay for the utilization of dental services from out-of-pocket resources. The increased dental visits by Afro-American duals seem to be delayed realization of their unmet needs for dental care.

## Discussion

Compared to Medicare-only beneficiaries, approximately 6.3 million dual eligibles in the U.S. are especially vulnerable and have high medical care costs [11]. They are repeatedly reported as poor and underserved population [12].

The dual eligibility program is designed to help low-income Medicare beneficiaries receive needed health care. As Medicare program's cost-sharing requirements – premiums, deductibles, and coinsurance – are often a financial burden to low-income beneficiaries and serve as a barrier to receiving needed care, federal and local governments have expanded over time Medicaid to certain eligibility groups whereby State Medicaid agencies are required to pay all or some of the Medicare out-of-pocket cost-sharing expenses for low-income Medicare beneficiaries that meet income and asset criteria [12]. Collectively, these individuals are referred to as *dual eligibles *or *duals*.

Dual eligibles qualify for Medicare because they are aged 65 or over, disabled and receive Social Security Disabled Income (SSDI) assistance younger than age 65. Dual eligibles qualify for Medicaid because they are aged, blind, or disabled and meet the income and asset requirements for Supplement Security Income (SSI) assistance. Additionally, medically needy individuals qualify for Medicaid because they spend down a large portion of annual income and assets to pay for their medical or long-term care costs [13]. Most of dual eligibles such as qualified Medicare beneficiaries (QMBs) and specified low-income Medicare beneficiaries (SLMB) are entitled to receive full Medicaid benefits. Others like qualifying individuals (OIs), however, are not entitled to full Medicaid benefits but subsidized for Medicare premiums and cost sharing [11].

In this paper, we use the nationally representative data from Medical Expenditure Panel Survey (MEPS) to explore the relationship between dual eligibility for Medicare and Medicaid, and health care utilization. Understanding dual eligibles – population, their health care needs and health care usage – is the goal of this study for contributing to development of a relevant health policy. It seems useful to look at racial sub-groups of dual eligibles because racial differences may provide a clue to understanding heterogeneous effects of dual eligibility on health care use (and potentially unmet needs) of the poor and underserved population.

Dually eligible beneficiaries are better off than low-income Medicare-only enrollees who do not have dual coverage because duals are entitled to additional health benefits from Medicaid. We find that dual eligibility is positively correlated with the likelihood of using and the frequency of home health days but it is not significantly correlated with the frequency of office-based physician visits and hospital inpatient nights at the 5% level. The frequency of dental visits is inversely correlated with dual eligibility.

The significantly large effect of dual eligibility on the likelihood of using and the frequency of home healthcare receipt days is remarkable over the whole sample and particularly among Asian sample with dual eligibility. The large effect of dual eligibility on the home healthcare use could be explained by the level and scope differences in home health benefits between Medicare-only and Medicare-Medicaid dual eligibility programs. The traditional Medicare-only program does not provide home health benefits after 60-days. Medicaid, however, as a part of the dual eligibility program, takes almost full financial responsibility for the use of home health care.

Two competing theories may be involved in interpreting the dramatically increased utilization of home health care by dual eligibles: (i) *over*utilization by duals under the Medicare-Medicaid dual eligibility program in response to the additional home health benefits from Medicaid and (ii) *under*utilization by non-duals under the Medicare-only program because of its stringent home health benefits.

With similar generosity of Medicare-only and dual eligibility program for office-based physician services, the likelihood of using and the frequency of physician visits are not correlated with dual eligibility at the 5% level. Without differences in the level of benefits for hospital care, dual eligibility is not correlated with the frequency of hospital inpatient nights though it is statistically significant to increase the likelihood of using hospital care at the 5% level. The additional dental benefit provided by Medicaid is even inversely correlated with the frequency of dental visits though it is not statistically significant to decrease the likelihood of using dental services at the 5% level. These results suggest that *over*utilization theory does not seem to be persuasive. Dual eligibles do not always increase utilization of services in response to expanded benefits as shown in dental benefits newly covered by Medicaid.

Increasing the likelihood of using home healthcare and the high incidence rate ratio (IRR) of home health use by duals relative to Medicare-only beneficiaries (Table 3) may be the result of delayed realization of their unmet needs under Medicare rather than the result of possible *over*utilization due to dual eligibility. The unmet need for home healthcare over the sample seems most urgent among all health care types examined by the study.

With respect to racial differences, dually eligible Afro-Americans use more office-based physician and dental services than white duals. Asian duals use more home health services than white duals at the 5% level. The dual eligibility program seems particularly beneficial to Afro-American duals. An epidemic like AIDS/HIV-positive, which is most prevalent among young Afro-American males, will not be treated under the traditional Medicare-only program while they are fully covered by Medicaid within the dual eligibility program [14,15].

It is also true that there may exist inverse causality running from healthcare needs to dual eligibility: high levels of healthcare needs and usage may lead to qualifying for Medicare and Medicaid. Unravelling this complex relationship, further studies would need to control for potential confounding factors that may affect the relationship between dual eligibility and healthcare utilization.

In addition, it would be interesting to conduct an analysis separately for the disabled (e.g., Medicare beneficiaries under 65) and the elderly (e.g., Medicare beneficiaries 65 or older). Inherent differences between the two sub-groups may have affected or attenuated some of the relationships estimated by the study. To better understand the utilization differentials between the dually eligible and Medicare-only beneficiaries, further efforts may be required for examining the disease/individual-specific utilization patterns of the health care to consider potentially inherent heterogeneity in the characteristics of the dually eligible beneficiaries and their non-dual counterparts.

## Conclusion

Dual eligibility has varied impact on health care utilization across service types. More utilization of home healthcare among dual eligibles appears to be the result of delayed realization of their unmet healthcare need under the traditional Medicare-only program rather than the result of *over*utilization in response to the expanded benefits of the dual eligibility program. The dual eligibility program is particularly beneficial to Asian and Afro-American duals in association with the provision of home healthcare and dental services.

## Notes

1. Another baseline count model would in any case have a higher proportion of zeros than the parent Poisson distribution [8]. One source of excess zeros in count data is *over*dispersion. Mullahy [16] emphasizes that the presence of excess zeros "is a strict implication of unobserved heterogeneity." In other words, "the existence of unobservable heterogeneity may be sufficient to explain excess zeros, without recourse to alternative specifications such as zero inflated or hurdle models [17]."

2. Vuong [18] has proposed a test statistic for *non*nested models in comparison with their alternative distribution. The logic of the testing procedure is to allow for *over*dispersion by specifying a negative binomial count data process, then examine whether, even allowing for the *over*dispersion, there still appear to be excess zeros [8]. Vuong's statistic is bi-directional. If the absolute value of the statistic is less than 2, then the test does not favour one model or the other. Otherwise, large values for one model (Model 1) whereas small (negative) values favour Model 2. Carrying out the test requires estimation of the two models (Model 1 and Model 2) and computation of the two sets of predicted probabilities.

3. The Poisson is the special case of the negative binomial with *α *= 0. Note that if *α *= 0 the negative binomial reduces to the Poisson. The null hypothesis *H*_0 _:*α *= 0 can be tested against the alternative *α *> 0 using the existing hypothesis test methods [8]. Under the assumption that relevant likelihood functions are specified, we have three classical statistical techniques for testing *over*dispersion hypotheses – the likelihood ratio (LR), Wald, and Lagrange multiplier (LM) tests [7,9,10]. A sound practice of testing the *over*dispersion hypothesis is to estimate both Poisson and negative binomial models. The likelihood ratio (LR) test uses -2 times the difference in the fitted log-likelihood of the two models [9]. Alternatively, a Wald test can be performed, using the reported *t *statistic for the estimated *over*dispersion parameter *α *in the negative binomial model [7]. For all of the outcome variables, the likelihood ratio (LR) test statistics (reported in Table 3) strongly reject the null hypothesis of the Poisson specification, indicating the presence of *over*dispersion.

4. Out of the 8,262 sample individuals, 88 respondents did not remember the amount of healthcare use. Specifically, we had 2, 7, 77, and 2 members without required information about healthcare use in office-based physician visits, hospital inpatient nights, home health receipt days, and dental visits. For computational precision, they were not included in calculating the means and standard errors.

## Competing interests

The author(s) declare that they have no competing interests.

## Authors' contributions

SM developed the idea and conducted the statistical analysis. JS completed the literature review. All authors contributed to the interpretation of the data and the preparation of the manuscript.

## Pre-publication history

The pre-publication history for this paper can be accessed here:



## Figures and Tables

**Table 1:** Descriptive statistics for Medicare-only beneficiaries (MOB) and dually eligible beneficiaries (DEB)

Variable	Total	MOB	DEB			
			
			Total	Asian	Black	Hispanic
N	8,262 (100%)	6,381 (77.2%)	1,881 (22.8%)	92 (1.11%)	475 (5.75%)	513 (6.21%)
Demographic variables						
Age (mean)	69.3 (15.4)	70.9*** (13.4)	64.1*** (19.8)	71.1 (16.0)	63.5 (20.7)	67.1 (18.1)
Elderly (age 65 and over)	0.80 (0.40)	0.84*** (0.37)	0.67*** (0.47)	0.88 (0.33)	0.65 (0.48)	0.77 (0.42)
Male	0.41 (0.49)	0.43*** (0.50)	0.32*** (0.47)	0.38 (0.49)	0.31 (0.48)	0.33 (0.42)
Married	0.41 (0.49)	0.47*** (0.50)	0.23*** (0.42)	0.44 (0.50)	0.15 (0.36)	0.31 (0.46)
Rural	0.27 (0.45)	0.26*** (0.44)	0.30*** (0.46)	0.04 (0.21)	0.28 (0.45)	0.38 (0.39)
College and more	0.12 (0.31)	0.13*** (0.33)	0.04*** (0.20)	0.12 (0.33)	0.03 (0.18)	0.02 (0.13)
High school graduation	0.40 (0.48)	0.40*** (0.49)	0.24*** (0.42)	0.12 (0.33)	0.25 (0.42)	0.12 (0.32)
Less than high school	0.48 (0.50)	0.47*** (0.50)	0.73*** (0.44)	0.76 (0.43)	0.74 (0.44)	0.86 (0.34)
Asian	0.02 (0.15)	0.02*** (0.13)	0.05*** (0.22)	--	--	--
Hispanic	0.17 (0.37)	0.13*** (0.34)	0.27*** (0.45)	--	--	--
Afro-American	0.18 (0.39)	0.16*** (0.37)	0.25*** (0.43)	--	--	--
Socioeconomic variables						
Poverty	0.25 (0.43)	0.19*** (0.39)	0.45*** (0.50)	0.33 (0.47)	0.53 (0.50)	0.38 (0.49)
Still work	0.11 (0.31)	0.13*** (0.34)	0.04*** (0.20)	0.02 (0.15)	0.03 (0.18)	0.03 (0.17)
Health-related variables						
Poor health	0.36 (0.48)	0.32*** (0.47)	0.50*** (0.50)	0.36 (0.48)	0.50 (0.50)	0.52 (0.50)
Medical condition(s) of priority list	0.48 (0.50)	0.48*** (0.50)	0.51*** (0.50)	0.36 (0.50)	0.58 (0.50)	0.51 (0.50)
Number of co-morbidities	5.67 (4.19)	5.44*** (4.01)	6.47*** (4.68)	4.15 (3.16)	5.80 (4.45)	6.40 (4.62)
Limitation(s) to ADL	0.11 (0.43)	0.09*** (0.42)	0.20*** (0.46)	0.14 (0.43)	0.22 (0.47)	0.22 (0.47)
Limitation(s) to IADL	0.19 (0.46)	0.15*** (0.45)	0.33*** (0.49)	0.24 (0.48)	0.36 (0.50)	0.30 (0.49)
Dependent variables						
Office-based physician visits	6.42 (8.26)	6.09*** (7.44)	7.54*** (10.52)	5.98 (17.94)	6.81 (11.43)	7.73 (10.05)
Hospital inpatient nights	1.95 (7.49)	1.73*** (7.02)	2.66*** (8.86)	0.24 (1.04)	2.73 (8.31)	2.80 (9.13)
Home health provider days	11.87 (51.4)	5.82*** (34.0)	32.5*** (84.7)	5.04 (27.3)	40.8 (90.3)	36.35 (96.9)
Total dental visits	0.78 (1.98)	0.87*** (2.10)	0.48*** (1.50)	0.39 (1.00)	0.45 (1.32)	0.51 (1.54)

Notes: Two-tailed t-tests are performed to see the statistical significance of differences in demographic, socioeconomic, health-related, and dependent variables between MOBs and DEBs. Mean values are reported with standard deviations in brackets. Mean values for the outcome variables reflect the mean values among all the eligible beneficiaries. Asterisks *, **, and *** indicate statistical significance at 10%, 5%, and 1% levels, respectively.

**Table 2:** Percentage of non-zero users and mean values for health services use among Medicare beneficiaries and non-zero users

**Health Services**	Medicare Beneficiaries	Non-Zero Users
Office-based physician visits	6.418 (100%)	7.474 (85.88%)
Hospital inpatient nights	1.945 (100%)	10.697 (18.18%)
Home health provider days	11.871 (100%)	103.058 (11.41%)
Total dental visits	0.783 (100%)	2.699 (29.00%)

Notes: *Medicare beneficiaries *represent all individuals that are covered by Medicare benefits. This group includes both users and non-users of healthcare services. *Non-zero users *indicate the respondents who had positive visits, nights, or days for each type of outcome variables. Mean values are measured for each type of outcome variables among *Medicare beneficiaries *and *non-zero users*, respectively. Numbers in the brackets indicate the percentage of each group out the whole sample.^4^

**Table 3:** Statistical association of dual eligibility and health care utilization

	Office-based physician visits (1)	Hospital inpatient nights (2)	Home health provider days (3)	Total dental visits (4)
Demographics				
Age	0.997 (0.001)***	0.997 (0.003)	1.003 (0.003)	0.991 (0.002)***
Male	0.993 (0.022)	1.380 (0.110)***	1.097 (0.105)	0.867 (0.044)***
Married	1.063 (0.023)**	0.768 (0.062)***	0.616 (0.066)***	1.178 (0.060)***
Less than High School	0.891 (0.020)***	0.979 (0.076)	0.969 (0.094)	0.778 (0.054)***
Rural	0.970 (0.023)	0.984 (0.077)	1.439 (0.133)***	0.759 (0.043)***
Region				
Northeast	1.083 (0.031)***	0.961 (0.100)	1.242 (0.150)*	1.357 (0.088)***
Midwest	0.962 (0.027)	0.974 (0.091)	0.807 (0.094)*	1.149 (0.074)**
West	0.901 (0.025)***	0.715 (0.070)***	0.799 (0.100)*	1.162 (0.075)**
Medical condition				
Poor Health	1.168 (0.026)***	1.292 (0.093)***	1.424 (0.125)***	0.757 (0.042)***
Co-morbidities	1.140 (0.003)***	1.060 (0.009)***	1.037 (0.010)***	1.081 (0.007)***
Chronic Disease	1.147 (0.023)***	1.120 (0.079)	0.850 (0.070)**	0.909 (0.043)**
Socioeconomic status				
Poverty	1.004 (0.025)	0.897 (0.074)	1.020 (0.092)	0.894 (0.075)
Still Work	0.908 (0.032)***	0.562 (0.079)***	1.602 (0.403)*	0.833 (0.062)**
Race & dual eligibility				
Asian	0.886 (0.087)	0.899 (0.458)	0.044 (0.034)***	0.510 (0.117)***
Hispanic	0.997 (0.036)	1.009 (0.132)	1.790 (0.313)***	1.082 (0.107)
Afro-American	0.959 (0.033)	1.063 (0.130)	2.216 (0.346)***	0.622 (0.072)***
Dual Eligibility	1.016 (0.037)	1.003 (0.112)	1.958 (0.234)***	0.708 (0.085)***
Dual*Asian	1.235 (0.184)	0.334 (0.238)	16.879 (15.54)***	1.621 (0.564)*
Dual*Hispanic	1.106 (0.067)*	1.193 (0.237)	0.943 (0.216)	1.253 (0.211)
Dual*Afro-American	1.170 (0.072)***	1.132 (0.226)	0.643 (0.132)**	1.926 (0.329)***

Inflation model = logit				
Log likelihood	-22573.77	-8603.889	-7757.978	-8696.873

Constant	-4.012 (0.912)***	1.480 (0.138)***	3.071 (0.201)***	-3.211 (0.840)***
Cancer	-22.16 (16771.5)	-0.500 (0.088)***	-0.418 (0.088)***	-0.235 (0.134)*
Diabetes	-20.73 (7065.7)	-0.518 (0.075)***	-0.456 (0.078)***	0.485 (0.102)***
Hypertension	-11.54 (54.42)	-0.158 (0.063)***	-0.029 (0.072)	0.043 (0.092)
Stroke	-1.228 (0.942)	-1.226 (0.129)***	-1.037 (0.115)***	0.404 (0.165)**
Depression	-1.314 (0.590)**	-0.269 (0.072)***	-0.352 (0.074)***	-0.136 (0.100)
Less than High School	0.769 (0.235)***	-0.094 (0.072)	-0.133 (0.081)*	1.084 (0.138)***
Poverty	-0.260 (0.266)	-0.314 (0.096)***	-0.167 (0.101)*	0.037 (0.146)
Still Work	0.404 (0.321)	0.084 (0.126)	0.943 (0.191)***	-0.742 (0.314)**
Asian	0.144 (0.945)	1.007 (0.340)***	1.130 (0.364)***	-0.391 (0.503)
Hispanic	0.130 (0.318)	0.152 (0.096)	0.274 (0.106)***	0.160 (0.148)
Afro-American	0.917 (0.255)***	0.321 (0.093)***	-0.026 (0.096)	0.030 (0.207)
Dual eligibility	-1.487 (0.761)*	-0.285 (0.082)***	-1.066 (0.082)***	0.241 (0.145)*
Income0	1.298 (0.987)	0.300 (0.189)	0.325 (0.272)	2.725 (0.813)***
Income1	1.614 (0.924)*	0.159 (0.146)	-0.511 (0.214)**	2.420 (0.795)***
Income2	1.194 (0.910)	-0.035 (0.136)	-0.620 (0.206)***	2.280 (0.790)***
Incoem3	0.861 (0.949)	0.211 (0.150)	-0.300 (0.225)	1.724 (0.780)**
LR Chi^2 ^(20)	2,766.11	136.25	199.10	333.40
Prob> Chi^2 ^(20)	0.000	0.000	0.000	0.000
Number of obs.	8,260	8,255	8,185	8,260
Nonzero obs.	7,094	1,501	942	2,395
Zero obs.	1,166	6,754	7,243	5,865
alpha	0.572 (0.014)	1.906 (0.170)	1.522 (0.090)	1.377 (0.119)
Likelihood-ratio (LR) test of alpha = 0:	Chibar^2^(01) = 1.7e+04 Prob>=Chibar^2 ^= 0.000	Chibar^2^(01) = 1.3e+04; Prob>=Chibar^2 ^= 0.000	Chibar^2^(01) = 8.3e+04; Prob>=Chibar^2 ^= 0.000	Chibar^2^(01) = 1457.18; Prob>=Chibar^2 ^= 0.000
Vuong test of ZINB vs. standard NB:	Z = 5.40; Prob>z = 0.000	Z = 7.76; Prob>z = 0.000	Z = 7.95; Prob>z = 0.000	Z = 8.21; Prob>z = 0.000

Notes: Zero-Inflated Negative Binomial (ZINB) regressions are performed. Incidence rate ratios and correlation coefficient estimates are presented for negative binomial (NB) and logit regressions, respectively. Corresponding exponential-scale and logit-scale standard errors reported in brackets. Categorized income variables are constructed by dividing family income by the applicable poverty line (based on family size and composition), with the resulting percentages grouped into 5 categories: negative or poor ("Income0", less than 100%), near poor ("Income1", 100% to less than 125%), low income ("Income2", 125% to less than 200%), middle income ("Income3", 200% to less than 400%), and high income ("Income4", greater than or equal to 400%). Two-tailed t-tests and one-tailed chi-square tests are applied to the estimated coefficients and model specification statistics (i.e., LR and Vuong). Asterisks *, **, and *** indicate statistical significance at 10%, 5%, and 1% levels, respectively.

## References

[B1] Center for Medicare and Medicaid Services (CMS) Office of Research Development and Information (2003). Profile of Medicare Dual Eligible Beneficiaries.

[B2] Komisar HL, Feder J, Gilden D (2000). The Role of Medicare and Medicaid in Financing Health and Long-Term Care for Low-Income Seniors. Commonwealth Fund Working Paper 375 Washington DC.

[B3] Ryan J, Super N (2003). Dual Eligible for Medicare and Medicaid: Two for One or Double Jeopardy?. National Health Policy Forum Issue Brief 794 Washington DC.

[B4] Health Care Financing Administration (1997). A Profile of Dually Eligible Beneficiaries.

[B5] Mullahy J (1986). Specification and Testing of Some Modified Count Data Models. Journal of Econometrics.

[B6] Lambert D (1992). Zero-Inflated Poisson Regression with an Application to Defects in Manufacturing. Technometrics.

[B7] Cameron AC, Trivedi PK (1998). Regression Analysis of Count Data.

[B8] Greene WG (2003). Economic Analysis.

[B9] Winkelmann R (2003). Econometric Analysis of Count Data.

[B10] Wooldridge JM, Pesaran MH, Schmidt P (1997). Quasi-Likelihood Methods for Count Data. Handbook of Applied Econometrics.

[B11] Miller EA, Weissert WG (2004). Managed Care for Medicare-Medicaid Dual Eligibles: Appropriateness, Availability, Payment, and Policy. Journal of Applied Gerontology.

[B12] Center for Medicare and Medicaid Services (CMS) (2004). Establishing a Balance on the Current Number of Dual Eligibles Washington DC.

[B13] Center for Medicare and Medicaid Services (CMS) (2004). List and Definition of Medicare/Medicaid Dual Eligibles Washington DC.

[B14] Williams PB (2003). HIV/AIDS Case Profile of African Americans: Guidelines for Ethnic-Specific Health Promotion, Education, and Risk Reduction Activities for African Americans. Family and Community Health.

[B15] Siegel K, Schrimshaw EW, Karus D (2004). Racial Disparities in Sexual Risk Behaviors and Drug Use among Older Gay/Bisexual and Heterosexual Men Living with HIV/AIDS. Journal of National Medical Association.

[B16] Mullahy J (1997). Heterogeneity, Excess Zeros, and the Structure of Count Data Models. Journal of Applied Econometrics.

[B17] Jones AM, Culyer AJ, Newhouse JP (2000). Health Econometrics. Handbook of Health Economics.

[B18] Vuong O (1989). Likelihood Ratio Tests for Model Selection and Non-nested Hypotheses. Econometrica.

